# Impact of Porcine Pancreas Decellularization Conditions on the Quality of Obtained dECM

**DOI:** 10.3390/ijms22137005

**Published:** 2021-06-29

**Authors:** Marta Klak, Ilona Łojszczyk, Andrzej Berman, Grzegorz Tymicki, Anna Adamiok-Ostrowska, Maciej Sierakowski, Radosław Olkowski, Andrzej Antoni Szczepankiewicz, Artur Kamiński, Agnieszka Dobrzyń, Michał Wszoła

**Affiliations:** 1 Foundation of Research and Science Development, 01-793 Warsaw, Poland; marta.klak@fundacjabirn.pl (M.K.); ilona.lojszczyk@fundacjabirn.pl (I.Ł.); andrzejberman@gmail.com (A.B.); grzegorz.tymicki@fundacjabirn.pl (G.T.); anna.adamiok@cmkp.edu.pl (A.A.-O.); 2 Polbionica Ltd., 01-793 Warsaw, Poland; 3 Medispace Medical Centre, 01-044 Warsaw, Poland; 4 Department of Biochemistry and Molecular Biology, Centre of Postgraduate Medical Education, 01-813 Warsaw, Poland; 5 Institute of Biological Sciences, Cardinal Stefan Wyszynski University in Warsaw, Wóycickiego 1/3, 01-938 Warsaw, Poland; m.sierakowski@uksw.edu.pl; 6 Department of Transplantology and Central Tissue Bank, Medical University of Warsaw, 02-004 Warsaw, Poland; rolkowski@wum.edu.pl (R.O.); artur.kaminski@wum.edu.pl (A.K.); 7 Laboratory of Electron Microscopy, Nencki Institute of Experimental Biology, Polish Academy of Sciences, 3 Pasteur Street, 02-093 Warsaw, Poland; szczepankiewicz@nencki.edu.pl; 8 Nencki Institute of Experimental Biology, 02-093 Warsaw, Poland; dobrzyn@nencki.edu.pl

**Keywords:** pancreas, decellularization, extracellular matrix, bioink, bioprinting, Triton-X100, detergent, DNA, tissue engineering

## Abstract

Due to the limited number of organ donors, 3D printing of organs is a promising technique. Tissue engineering is increasingly using xenogeneic material for this purpose. This study was aimed at assessing the safety of decellularized porcine pancreas, together with the analysis of the risk of an undesirable immune response. We tested eight variants of the decellularization process. We determined the following impacts: rinsing agents (PBS/NH_3_·H_2_O), temperature conditions (4 °C/24 °C), and the grinding method of native material (ground/cut). To assess the quality of the extracellular matrix after the completed decellularization process, analyses of the following were performed: DNA concentration, fat content, microscopic evaluation, proteolysis, material cytotoxicity, and most importantly, the Triton X-100 content. Our analyses showed that we obtained a product with an extremely low detergent content with negligible residual DNA content. The obtained results confirmed the performed histological and immuno-fluorescence staining. Moreover, the TEM microscopic analysis proved that the correct collagen structure was preserved after the decellularization process. Based on the obtained results, we chose the most favorable variant in terms of quality and biology. The method we chose is an effective and safe method that gives a chance for the development of transplant and regenerative medicine.

## 1. Introduction

Each year, the shortage of organs for transplantation condemns many patients to arduous substitution treatment, severe complications, and consequently, even death [1]. Although transplants from deceased donors are often the only way for patients to return to everyday life, such complicated surgical procedures are often associated with numerous postoperative complications and the possibility of rejection of the transplanted organ by the recipient’s immune system [2,3]. Therefore, tissue engineering is a promising field in the reconstruction and replacement of missing or malfunctioning tissues and organs [2,3,4,5].

One of the branches of tissue engineering that is currently thriving is 3D bioprinting. This pioneering technology allows the production of biomimetic constructs with a heterogeneity of tissue composition [6,7]. Despite the large variety of natural and synthetic biomaterials (such as collagen, alginate, and polyglycolic acid), it is still challenging to mimic the complexity of native tissues [8,9]. The extracellular matrix (ECM) is a naturally occurring scaffold secreted by the resident cells of each tissue and organ, which forms a cellular microenvironment composed of glycoproteins, collagens, glycosaminoglycans, and proteoglycans [10]. ECM ensures the shape and strength of many tissues, provides optimal conditions for the functionality of the cells included in the organ, and is an essential source of growth factors [11,12]. ECM proteins (collagens, proteoglycans, and glycoproteins) and their spatial structures can determine cell behavior and viability through communication with the intracellular cytoskeleton [13,14].

In recent years, decellularized extracellular matrices (dECMs) have been recognized as one of the most promising biocomponents that can be used in the 3D-bioprinting process, diagnostics, and regenerative medicine [15]. dECMs retain functional and structural proteins and bioactive indicators that are included in natural ECM [16,17]. Rivetingly, it has been demonstrated that manipulating dECMs by various techniques is an efficient strategy of controlling and imparting new dECM characteristics. Such dECMs can be used in many therapeutic applications (e.g., muscle, neural tissue and liver regeneration, vascular grafts, cartilage repair, insulin delivery, skin grafts) [18].

The most common factors used in the decellularization process of tissues and whole organs are ionic (SDS) and/or non-ionic (Triton X-100) detergents [19,20]. They are considered the most effective means in the decellularization process. However, the results of many studies indicate a lower degree of damage to the ECM components using Triton X-100 [19,21,22,23].

It is worth noting that all factors (e.g., detergent, flushing agent) and protocols (e.g., duration/temperature of the process) used for tissue decellularization can change the composition of dECM and cause disruption to its microarchitecture [24,25]. Therefore, this study aimed to assess how the conditions of the decellularization process affect the quality of the final product. For this purpose, we analyzed the influence of the degree of disintegration of the tissue material, the type of rinsing agent, and the temperature on the protein composition, the content of residual DNA, fat, and most importantly, the amount of remaining detergent.

So far, in the literature [19], an effective decellularization process has been determined based on the residual DNA content and histological evaluation. However, the evaluation of the concentration of detergent remaining in the decellularized tissue is not a standard part of the research reported in the art and is practically never conducted. The assessment of the residual detergent content in dECM is difficult, but from our point of view it is necessary due to the high cytotoxicity of Triton X-100. It should be also emphasized that too high residual content of Triton X-100 in dECM will prevent the use of such material in clinical trials [26]. It seems that the success of the decellularization process should be based as much on the removal of genetic material as the removal of the detergent. These two parameters should, first and foremost, prove the quality of the entire process. These research approaches are not widely described in the literature, wherefore we developed a new way for determining the content of triton X-100 in dECM and conducted cytotoxicity tests to confirm the biological quality of decellularized tissue.

## 2. Results

### 2.1. The Amount of Residual DNA Concentration

**Figure 1:**
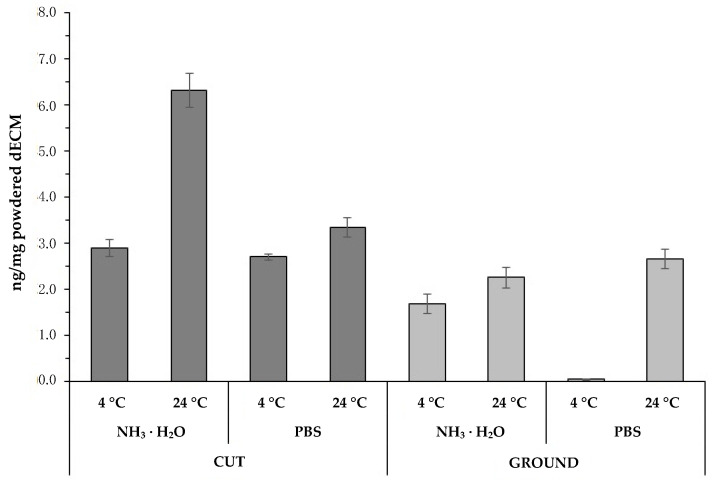
The graph shows the results of residual DNA in the final product after decellularization, lyophilization, and milling on a cryogenic mill. In the event of the grounded tissue, the PBS solution at 24 °C turned out to be the least effective. All other tests showed significantly lower residual DNA values. As in the case of the PBS solution, a significant decrease in DNA content was demonstrated for the NH_3_ · H_2_O solution at 4 °C vs. 24 °C.

Quantitative Pico-Green analysis for residual DNA content in the decellularized material showed that it was virtually completely removed relative to native tissue. The method of preparing the material for the decellularization process significantly affected the final concentration of genetic material in the obtained dECM. Values below the generally accepted 50 ng/mg standard [19] were obtained in all tested samples. However, in tissue subjected to the grinding process, statistically lower residual DNA values were found compared to the cut tissue (Figure 1). Samples that were cut into small fragments during material preparation stage showed a residual DNA content ranging from 2.71 ± 0.07 ng/mg (PBS, 4 °C) to 6.32 ± 0.37 ng/mg (NH_3_ · H_2_O, 24 °C) in powdered dECMs. However, in the case of material mechanically disintegrated by means of a machine, the amount of remaining genetic material was significantly lower and amounted from 0.05 ± 0.07 ng/mg (PBS, 4 °C) to 2.65 ± 0.21 ng/mg (PBS, 24 °C). It is worth noting that the lowest residual DNA values were obtained, regardless of the grinding method, using PBS as a rinse agent and the temperature of 4 °C.

The obtained residual DNA results were on average more than 17 times lower than the norm. Moreover, the values received for all analyzed variants were over 320 times lower than the results achieved for the native tissue, i.e., 811 ± 155 ng/mg (data not shown in the diagram).

Next, the impact of the scrubbing solution used during the decellularization process was analyzed. The analysis was carried out on samples that were milled at the stage of processing the tissue material. Based on the analyses carried out, it has been shown that both the type of fluid used and the temperature at which the entire process is carried out is significant for the final content of genetic material in the obtained raw material (Figure 1). Both when using the PBS solution and NH_3_ · H_2_O, it was shown that a significant reduction in residual DNA content occured when the process is carried out at 4 °C.

**Figure 2:**
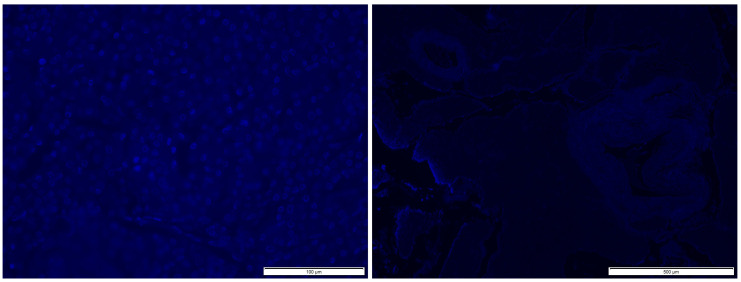
Microscopic image of DAPI staining of native tissue ((**a**); scale: 100 µm) and decellularized tissue ((**b**); scale: 500 µm).

DAPI staining performed for the most preferred variant of the decellularization process (ground tissue/PBS as rinse agent/4 °C) showed no cell nuclei (blue structures in Figure 2a) in the decellularized tissue (Figure 2b). This examination confirmed the results obtained with the PicoGreen assay (Figure 1).

### 2.2. Proteomic Analysis of Powdered dECM

**Figure 3:**
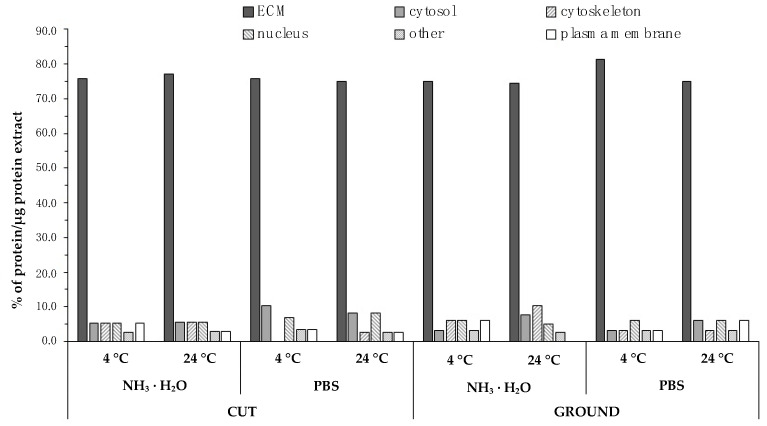
Percentage of proteins in the final decellularization product depending on the method of tissue disintegration (cut vs. ground), temperature (4 °C vs. 24 °C), and the solution used for rinsing (NH_3_ · H_2_O vs. PBS).

Protein composition analysis showed that, regardless of the decellularization protocol used, over 70% of proteins derived from the extracellular matrix were found in all evaluated samples (Figure 3; Table 1).

It should be noted, however, that when PBS was used as the scrubbing solution, tissue was ground, and the temperature of the entire process was maintained at 4 °C, the ECM protein content was the highest. In detail, in this variant, more than 80% of the total protein content was made up of ECM proteins (most of which is collagen) and less than 20% was other proteins (nuclear proteins, resulting from the breakdown of cell organelles, cell membrane, cytosol or cytoskeleton of cells building the pancreas organ).

**Figure 4:**
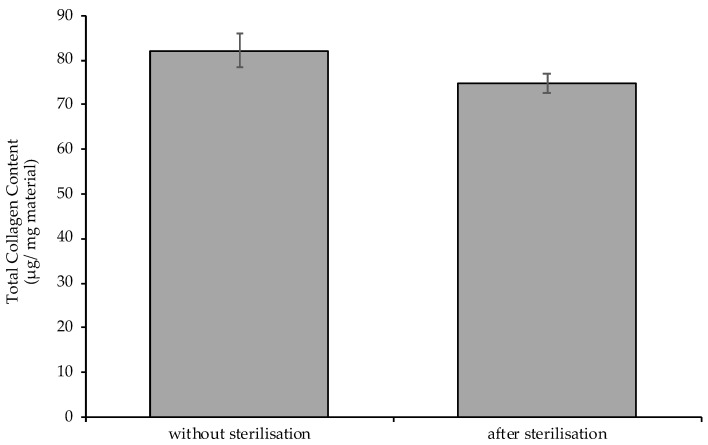
Total collagen content in dECM powder obtained with the use of the most preferable variant (i.e., ground/PBS/4 °C) without sterilization (i.e., before sterilization; left graph bar) and after sterilization (right graph bar); MV ± SD, *n* = 4, *p* = 0.0230.

ECM samples were sterilized by radiation. The literature has reported the influence of this sterilization method on the composition of the sterilized material [27,28,29]. Therefore, it was decided to analyze the total collagen content of the ECM powder, obtained with the use of the most preferable variant (i.e., ground/PBS/4 °C), subjected and not subjected to radiation sterilization (Figure 4). As expected, a decrease in the total content of this fibrillar protein was observed (without sterilization, 82.2 ± 3.7 μg/mg; after sterilization, 74.8 ± 2.1 μg/mg). However, after the sterilization process, collagen content in the ECM powder produced according to our method proposed in this article was still very high.

### 2.3. Contents of the Remaining Triton X-100 Detergent in the Final Product

**Figure 5:**
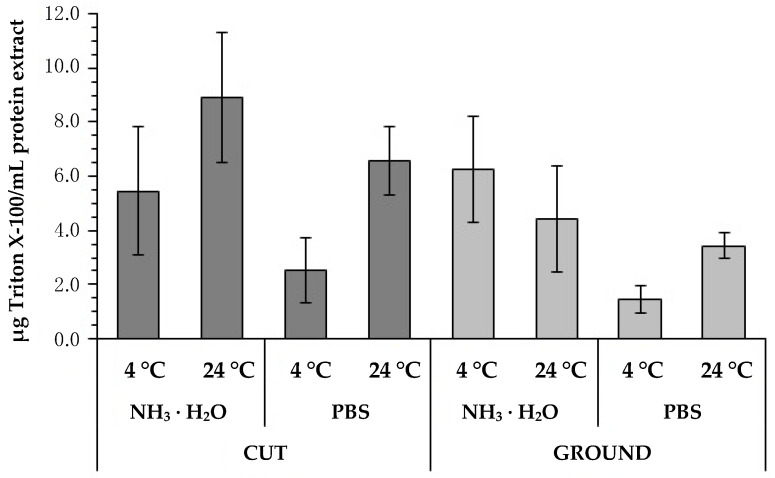
Evaluation of residual detergent content in the final product (i.e., in dECM powder) depending on how the tissue was minced (cut vs. ground), the solution used for rinsing (NH_3_ · H_2_O vs. PBS), and temperature (4 °C vs. 24 °C).

Evaluation of the final content of the used detergent conducted by our novel manner showed significantly lower values when using the method based on grinding the material in preparation for the decellularization process (Figure 5). In these samples, the remaining detergent level was lower in cut material (cut vs. ground: 5.47 ± 2.38 μg/mL (4 °C, NH_3_ · H_2_O), 8.91 ± 2.38 μg/mL (24 °C, NH_3_ · H_2_O), 2.54 ± 1.19 μg/mL (4 °C, PBS), 6.59 ± 1.26 μg/mL (24 °C, PBS) vs. 6.26 ± 1.96 μg/mL (4 °C, NH_3_ · H_2_O), 4.42 ± 1.96 μg/mL (4 °C, NH_3_ · H_2_O), 1.46 ± 0.51 μg/mL (4 °C, PBS), 3.44 ± 0.50 μg/mL (24 °C, PBS)). In addition, the effect of the rinse agent on the amount of detergent remaining was analyzed (Figure 5; Table 2). Also, in this case, the advantage of using PBS solution for leaching Triton X-100 was proved. In the samples where this solution was used, lower concentration of the remaining detergent was observed.

### 2.4. Total Fat Content

**Figure 6:**
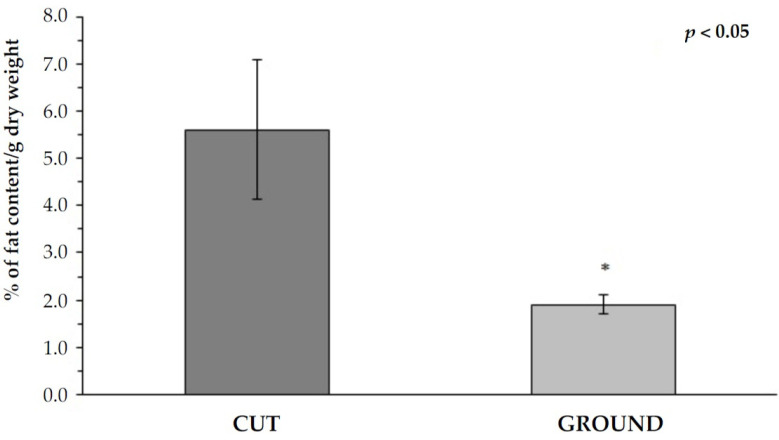
The percentage of total fat depending on the method of grinding the tissue undergoing the decellularization process (MV ± SD, *n* = 3, * *p* < 0.05).

The fat content assessment in the final product significantly depended on the initial preparation of the tissue material (Figure 6). The tissue, which at the initial stage of treatment was cut with small scissors into small fragments, in the final product, showed 2.95 times higher total fat content than ground tissue (cut vs. ground: 5.6% vs. 1.9%; *p* < 0.05) and two times lower than native tissue (11.6%—data not shown in Figure 6).

### 2.5. Histological Analysis

**Figure 7:**
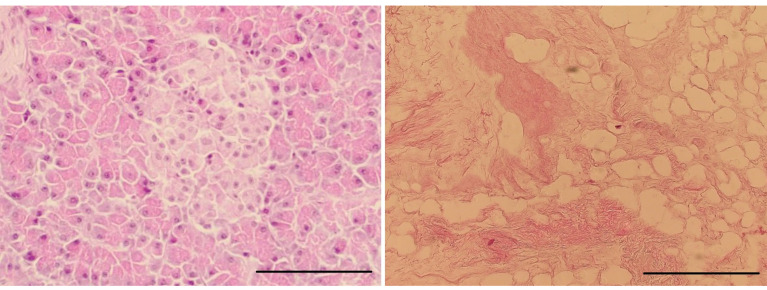
Elimination of nuclear material after decellularization and histological evaluation of decellularized pancreas—control tissue ((**a**); 100 µm; magnification 6.3 kx) and decellularized tissue ((**b**); 100 µm; magnification 6.3 kx).

**Figure 8:**
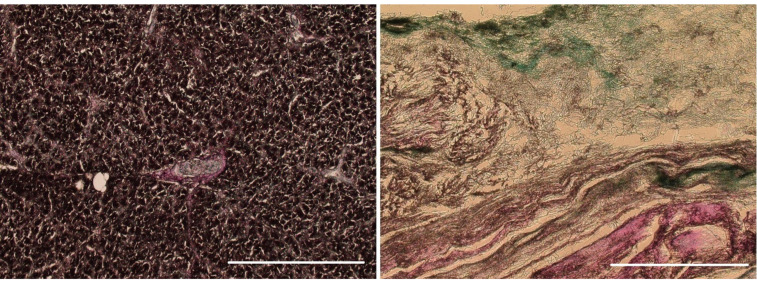
Microscopy images of native tissue ((**a**); 100 µm; magnification 6.3 kx) and decellularized tissue ((**b**); 100 µm; magnification 6.3 kx) stained with Alcian blue and van Gieson.

**Figure 9:**
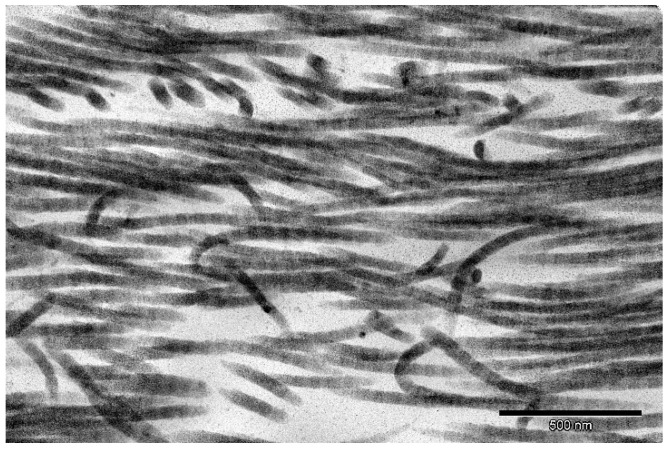
TEM image of decellularized pancreas (magnification 80 kx).

H + E staining confirmed the decellularization process’s efficiency, which resulted in the complete removal of cellular material while maintaining a structure comparable to native tissue (Figure 7). Cellular elements, including nuclei (purple structures), were visible in the native tissue (Figure 7a). These structures were not present in the decellularized tissue (Figure 7b), which confirms the effectiveness of the proposed decellularization method. Moreover, Alcian blue–van Gieson staining of native tissue (Figure 8a) and decellularized tissue showed the presence of GAGs (blue–green structures on Figure 8b), and collagen in decellularized tissue (mauve structures on Figure 8b)—those structures were also clearly visible on the TEM image (Figure 9).

### 2.6. SEM/TEM Analysis

**Figure 10:**
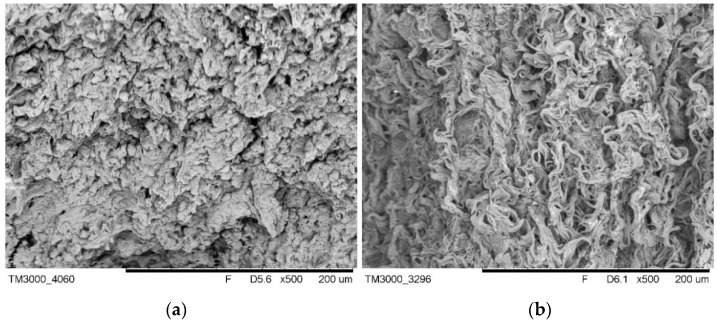
SEM image of native ((**a**); magnification 500×) and decellularized ((**b**); magnification 500×) pancreas.

Comparison of the SEM of native (Figure 10a) and acellular (Figure 10b) pancreas showed the preservation of the three-dimensional microstructure after decellularization with clearly visible ECM protein fibers. Furthermore, the TEM image confirmed the preservation of proper collagen fibril structure (characteristic bands) after the decellularization process (Figure 9).

### 2.7. Cytotoxicity Evaluation

**Figure 11:**
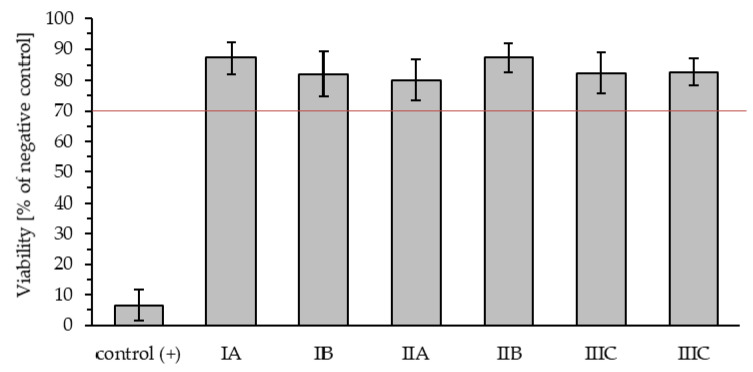
The viability of the L929 cells cultured with extracts of the dECM–1 mg/mL (MTT assay, MV ± SD, *n* = 4).

In compliance with ISO 10993-5 standard, a material is non-cytotoxic if cell viability does not drop by 30% compared to the negative control. Viabilities of L929 cells (mouse fibroblasts) after 24 h-culture with extracts were about 80–87%, wherefore according to ISO 10993–5, all samples (from three different series in two repetitions, labeled as IA, IB, IIA, IIB, IIIA, IIIB) were shown to be non-cytotoxic (Figure 11).

None of the tested samples did exhibit cytotoxicity, which leads to the conclusion that the presented decellularization process is repeatable and enables obtaining non-cytotoxic products. Additionally, MTT test results (which did not show a significant difference between samples–87 ± 5% for IA, 82 ± 7% for IA, 80 ± 7% for IIA, 87 ± 5% for IIB, 82 ± 7% for IIIA and 83 ± 4% for IIIB) confirmed results received during the assessment of the remaining detergent contents in the final product.

## 3. Discussion

The production of tissue models using the latest technologies (including 3D bio-printing) enables the use of bionic organs and tissues to regenerate those damaged inpatients or to replace them completely. Of course, it will be a few more years before bioprinting becomes a permanent feature of clinical practice. However, the research and results achieved by scientists worldwide allow us to be optimistic about the further development of this scientific field. The potential benefits of using biological materials based on dECM to replace and rebuild damaged or non-functional tissues and organs are noteworthy. Of course, you can use various types of materials for biodegradation, both synthetic and natural. However, dECM obtained from native organs and tissues is characterized by a decisive advantage. One of the most common methods used to obtain fines of the extracellular matrix from tissues is the decellularization process. However, despite the many available protocols, a universal decellularization method cannot be clearly identified for each source tissue type.

In this study, we focused on testing various conditions (tissue fragmentation, temperature, flushing agent) during the pig pancreas’ decellularization process in order to assess the quality of the resulting dECM. We decided to use a scrub with Triton X-100 non-ionic detergent, followed by the use of DNase enzymes to remove nucleic acid residues. Triton X-100 is a detergent capable of interfering with lipid–lipid and lipid–protein interactions, leaving intact protein–protein interactions, suggesting the legitimacy of using this detergent to obtain biocompatible scaffolds [30,31,32].

Despite the fact that detergents such as Triton X-100 are extremely effective in the decellularization process, it should be remembered that the detergent residual concentration and toxicity remain the main problems in this process. Cytotoxicity is possible even at reduced agent concentrations, and the main toxic effect of this detergent is its cytolytic and hematolytic activity, resulting from impaired cell membrane integrity, mitochondrial function, and cellular metabolism [26,33,34]. Triton X-100 as a surfactant is used in the production of vaccines, and its acceptable amounts are from 0.17 to 0.5 mg/mL [35]. The test results showed the effectiveness of our protocol, resulting in dECM with a residual detergent content below the cytotoxicity level, and the lowest results for this parameter were recorded in the group subjected to tissue grinding and using 1×PBS as a washing agent (Figure 5). Additionally, the MTT tests performed by us for the aforementioned most favorable variant confirmed the lack of cytotoxicity of the obtained dECM powder. Moreover, other scientists have also proven that a non-cytotoxic product can be received by decellularization using Triton X-100 [36].

An equally important subject of research was the nucleic acid residues remaining in the dECMs. Remains of DNA fragments can cause problems with immunological compatibility. The best solution is to use xenogenic material. The advantage of this solution is, for example, easy access to biological material, and thus there is also a significant production potential. However, in the case of choosing such a path of obtaining a dECM, the potential immunogenicity should be sufficiently reduced [37]. Therefore, the goal is to obtain DNA below 50 ng/mg dsDNA in the final material, and the DNA fragment length should not exceed 200 bases [19,38]. Our dECM also met these standards because the amount of isolated DNA in each analyzed variant was much lower than the recommended standard [19]. In addition, we found that grinding the tissue by milling and using a 1×PBS solution at 4 °C as a rinse agent after the detergent had a significant effect on the removal of residual DNA content. Such results confirmed the literature reports showing the removal of more than 90% of the genetic material during the decellularization process with the use of Triton-X100 [39,40,41,42,43,44,45,46].

Literature reports have shown that Triton X-100 affects the removal of glycosaminoglycans (GAG) [47] and retention of cellular debris [48]. Such results were presented by a group of scientists engaged in the decellularization of the aortic valve [47]. In turn, our histological analysis of pancreatic tissue [49], which we decellularized, confirmed the effectiveness of Triton X-100 as a decellularizing agent, which resulted in the absence of visible cellular debris while maintaining the architectural structure of the tissue, including GAG. Moreover, the proteomic analysis of the obtained dECM showed that in all the used washing variants, the main remaining components were ECM proteins, with a definite predominance of collagen. Therefore, the selection of appropriate process parameters is crucial to achieving a material that reflects the native tissue as much as possible.

In this article, we have developed a massive tissue decellularization method. We proposed eight variants of the decellularization process (we analyzed the influence of different rinsing agents, different temperatures, and methods of grinding native material). Based on the results of detailed studies obtained, we chose the most preferred variant, which was additionally analyzed (e.g., cytotoxicity tests). We evaluated the residual Triton X-100 content in the decellularized tissue, even though this assessment is not a standard part of the research reported in the existing literature and is practically never conducted. Our analyses have shown that we retreived a product with an extremely low Triton X-100 content with negligible residual DNA content. It should also be foregrounded that the very low residual content of Triton X-100 in our dECM may allow the use of this material in clinical trials. Moreover, performed stainings (Alcian blue–van Gieson, DAPI, and Harris hematoxylin–Eosin stainings) confirmed the obtained results. Furthermore, the TEM microscopic analysis proved that the correct collagen structure was preserved after the decellularization process. To sum up, the manner we propose is a repeatable and effective method; moreover, it evolved to European Patent Application (EP19218191.5) and International Patent Application (PCT/IB2020/056856).

## 4. Materials and Methods

**Figure 12:**
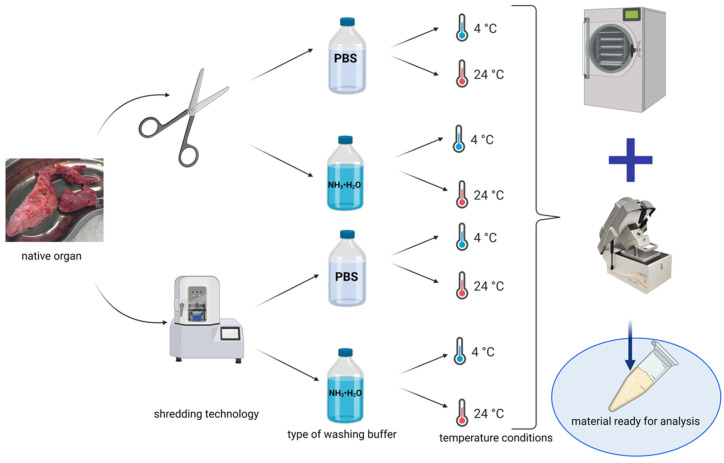
Experiment scheme including method of preparation of biological material. Created in BioRender.com, accessed on 27 June 2021.

Pig pancreases with a total weight of about 5 kg were used for the decellularization process. The material was collected in a local slaughterhouse. All organs were frozen at −20 °C after collection. Then they were thawed and cleared of fat. The pancreas was immersed in PBS (Tablets, Takara, Janki, Poland)/streptomycin (Sigma Aldrich/Merck; Warsaw, Poland) solution (final concentration 0.01% *v*/*v*) before the actual experiment. The cleaned pancreatic material was divided into eight parts equal in mass. As part of the experiment, temperature conditions, type of flushing agent, and method of grinding the tissue material were tested (Figure 12). A 1% Triton X-100 (Sigma Aldrich/Merck; Warsaw, Poland) solution with 0.1% NH_3_ · H_2_O in 1× concentrated PBS was used as the detergent in each case. All tested variants were placed in the incubator (Eppendorf Innova^®^ 42R, Warsaw, Poland) throughout the experiment (maintaining the appropriate temperature variants) with constant stirring of 150 rpm.

The decellularization process lasted five days, and during this time, the detergent was changed every 4 h for the first three days and every 24 h for the next two days. The detergent was then rinsed for 72 h with 0.1% NH_3_ · H_2_O or 1×PBS solution (according to the experimental design). The penultimate step was the addition of a 0.0002% DNase solution in 1×PBS with 0.12 mM calcium and magnesium ions. This stage lasted 8 h and was conducted at 37 °C regardless of the whole process’s analyzed conditions. The last step was to rinse all variants again with a 1×PBS solution. All solutions used for decellularization were enriched with streptomycin at a final concentration of 0.01%.

After the process was completed, the obtained extracellular matrix was frozen in liquid nitrogen and ground into fragments about 0.5 cm in size. The material prepared in this way was subjected to a freeze-drying process. The lyophilizate thus obtained was ground into a powder with the use of a cryogenic mill (SPEX^®^ SamplePrep 6775, Rickmansworth; UK). All obtained samples were subjected to radiation (25 kGy) to sterilize the material.

### 4.1. DNA Evaluation

Total DNA was extracted from porcine tissues using DNeasy Blood & Tissue kit (Qiagen, Hilden, Germany) in accordance with manufacturer’s instruction. Determination of DNA purity was performed by absorbance spectroscopy. Thus, the absorbance of DNA samples was measured at 260 and 280 nm with BioTek Synergy H1 microplate reader (Winooski, VT, USA) in order to calculate the A260/280 ratios.

To determine the residual content of genetic material (DNA) in the analyzed powdered dECM, commercially available genomic DNA isolation kits (DNeasy Blood & Tissue Kit from Qiagen) and the Pico Green kit (Quant-iT PicoGreen dsDNA from Molecular Probes, Life technologies, Foster City, CA, USA) were used. The procedures were carried out following the manufacturer’s instructions attached to both sets. Quantitative analysis of genetic material was performed using a Synergy H1 Hybrid Multi-Mode Microplate Reader (BioTek, Winooski, VT, USA).

Native tissue samples and decellularized tissue samples were fixed with 4% paraformaldehyde (PFA, Sigma-Aldrich/Merck, Warsaw, Poland) and stained using DAPI (blue staining of a nucleus, Invitrogen, Warsaw, Poland) according to the protocol provided by the manufacturer and analyzed using fluorescent microscopy.

### 4.2. Mass Spectrometry Analysis

Protein extraction was carried out in RIPA buffer, then to determine the protein concentration in the samples, BCA (Pierce™ BCA Protein Assay Kit) analysis was performed. Standardized amounts of proteins (40 µg protein) were loaded onto a polyacrylamide gel, and electrophoretic separation of proteins was carried out in a constant intensity field. After the electrophoresis, the entire gel was stained with BlueStain Sensitive Plus (EURx, Gdansk, Poland). The prepared samples were subjected to MS analysis at Harvard University (Harvard Medical School, Taplin Mass Spectrometry Facility, Boston, MA, USA) according to the procedures used in the center’s procedures. The analysis of the obtained results was based on the UniProt database.

Moreover, the total collagen content in the ECM powder was assessed by performing the analysis using the Total Collagen Assay Kit (ab222942, Abcam, Cambridge, UK) according to the manufacturer’s instruction. Samples of ECM powder produced by the variant evaluated as the most preferred, without and after sterilization, were tested.

### 4.3. Assessment of the Content of Detergent Used in the Final Product of the Process

To assess the amount of detergent remaining in the final product (powdered dECM), the powder was dissolved using collagenase (NB 8 Broad Range; Nordmark, Uetersen, Germany) at a concentration of 20× (compared to the manufacturer’s recommended concentration/g of tissue) for 24 h. Triton X-100 quantification was conducted spectrophotometrically by the potassium salt of tetrabromophthalein ethyl ester. The TBPE-K reagent method was used for low ethoxylated non-ionic surfactants. It involves the formation of a colored non-ionic surfactant complex with TBPE-K reagent (Alfa Aesar, Kandel, Germany) in a slightly alkaline medium. After extracting the complex with dichloromethane (CHROMASOLV from Honeywell), the extract’s absorbance was measured (Shimadzu UV-2700 spectrophotometer, Duisburg, Germany).

In the first step, the samples were diluted with water. Then 2.5 mL of the buffered TBPE-K reagent and 0.5 mL of 0.1 M KOH (Sigma-Aldrich/Merck, Warsaw, Poland) were added to 2.5 mL of the sample and mixed thoroughly. The entire procedure was carried out in accordance with the manufacturer’s instructions. Samples were left for 2 min. After this time, 5 mL dichloromethane (DCM; Sigma-Aldrich/Merck, Warsaw, Poland) was added and shaken for 2 min. Phases were left to separate. The lower DCM phase was collected, and absorbance at 620 nm was measured. The calibration curve was prepared from Triton X-100 in the concentration range 1–15 mg/L (calibration curve formula: y = 0.0225x + 0.049; R^2^ = 0.9956).

### 4.4. Total Fat Content Assessment

The fat content was determined using the classic Soxhlet method using petroleum ether extraction. The extraction was carried out for 6 h at the solvent temperature within 70 °C. Quantitative analysis of fat contained in the tested samples was carried out on the basis of differences in the weight of the thimble before and after the extraction process.

### 4.5. Histological Analysis

The material after decellularization was fixed in a 4% paraformaldehyde solution. In the next stage, the test material was washed in water, dehydrated, and X-rayed using a tissue processor (Microm STP 120, Microm International GmbH, Dreieich, Germany) using Ottix Shaper and Ottix Plus reagents (Diapath, Microstain Division, Via Savoldini, Martinengo, Italy). The samples were impregnated in a paraplast^®^ (Sigma-Aldrich/Merck, Warsaw, Poland), and then embedded in paraffin in the form of blocks, which were cut on a rotary microtome (Microm HM 355, Microm International GmbH, Dreieich, Germany) into sections of 4 μm thickness. Preparations adhered to slides (SuperFrost Ultra Plus, Thermo Scientific, Waltham, MA, USA) were dewaxed and stained with Harris hematoxylin (Thermo Fisher Scientific, Waltham, MA, USA) and eosin (Sigma-Aldrich/Merck, Warsaw, Poland) and then closed using synthetic DPX resin (Fluka Chemie GmbH, Buchs, Switzerland). Staining was performed to visualize protein structures and residues of genetic material in the form of cell nuclei.

Native tissue samples and decellularized tissue samples were fixed in 10% formalin at 5 °C for 24 h and dehydrated at room temperature using alcohol solution with increasing alcohol concentrations, and finally embedded in paraffin. The samples were cut, adhered to glass slides, and dried at 60 °C for 1 h. The materials were rehydrated. Alcian blue–van Gieson (Abcam, Cambridge, UK) staining was performed according to the protocol provided by the manufacturer.

### 4.6. SEM Evaluation

Samples of the material were washed with phosphate-buffered saline (PBS, Thermo Fisher Scientific), cut in 2 mm thick slices, and fixed. Fixation was done in a solution of 2.5% glutaraldehyde (Sigma-Aldrich/Merck, Warsaw, Poland) in sodium cacodylate buffer (Sigma-Aldrich/Merck, Warsaw, Poland) for 15 min at room temperature. Then, samples were consecutively washed in sodium cacodylate buffer, distilled water, and 70% ethanol, 15 min in each solution. Thereafter samples were dehydrated in graded alcohol series for 10 min each: 80% ethanol, 90% ethanol, 100% ethanol.

The samples were set aside to dry at room temperature. Then, samples were coated with gold in a Leica EM SCD050 sputtering device (Leica Microsystems, Wetzlar, Germany). SEM observation was performed in TM3000 scanning electron microscope (Hitachi High-Technologies, Tokyo, Japan).

### 4.7. TEM Evaluation

The tissue was fixed using 2.5% glutaraldehyde (Sigma-Aldrich/Merck, Warsaw, Poland) and 2% paraformaldehyde (Sigma-Aldrich/Merck, Warsaw, Poland) solution at 4 °C overnight. After fixation, the material was rinsed three times (10 min) in 0.1 M cacodylate buffer. Subsequent, small tissue fragments (approximately 1 mm^3^) were postfixed in 1% osmium tetroxide for 1 h at room temperature. Dehydration was conducted by incubating the analyzed sample in solution of increasing ethanol concentrations (50%, 70%, 90%, 96%, and 100%), and then in a mixture of ethanol and propylene oxide (1:1), and in pure propylene oxide. During dehydration, samples were stained using 1% uranyl acetate in 70% ethanol. Lastly, materials were embedded in the Epon resin. Ultrathin sections were collected on TEM grids and poststained using uranyl acetate and Reynold’s lead citrate. Electron micrographs were obtained with a Morada camera on a JEM 1400 transmission electron microscope at 80 kV (JEOL Co., Tokyo, Japan) in Laboratory of Electron Microscopy, Nencki Institute of Experimental Biology of Polish Academy of Sciences, Warsaw, Poland.

### 4.8. Cytotoxicity Evaluation

To assess the cytotoxicity of the dECM, an MTT test was conducted. Firstly, in 96-well plates (for cell culture) with a flat bottom, mouse fibroblasts (L929 cells; Sigma Aldrich) were seeded with a concentration of 10^4^ cells/100 μL culture medium/well. The culture medium (Dulbecco’s modified Eagle medium without phenol red, Gibco, Warsaw, Poland) contained fetal bovine serum (FBS; 10%, Gibco, Warsaw, Poland), l-glutamine (Gln, 1%, Gibco, Warsaw, Poland), and penicillin/streptomycin (Pen/Strep; 1%, Gibco, Warsaw, Poland). To make extracts, sterile dECMs were incubated with supplemented cell-culture medium (1 mg/mL) for 24 h at 37 °C, 5% CO_2_. The next day, fibroblasts in 96-well plates were washed with phosphate-buffered saline without Mg^2+^ and Ca^2+^ and then put in contact with extracts (100 μL/well) for 24 h at 37 °C, 5% CO_2_. As a negative control–control (−), cells cultured in supplemented culture medium without contact with the analyzed samples throughout the entire test were used. As a positive control–control (+), cells treated with 1% Triton X-100 detergent solution were used. Triton X-100 in high concentrations is known to be cytotoxic. This check is necessary to confirm the proper conduction of the experiment. The following day, all extracts were collected from plates, and subsequently, cells were washed with DPBS without calcium and magnesium ions. Lastly, 50 μL of MTT solution (Sigma-Aldrich) was added to each well. Then, 96-well plates were incubated for 4 h at 37 °C, 5% CO_2_. After that, using a spectrophotometric microplate reader (BioTek, Winooski, VT, USA), the absorbance of the samples was measured at 570 nm (reference wavelength: 650 nm).

### 4.9. Statistical Analysis

Data are presented as mean ± standard error (SD) unless otherwise indicated. All statistical analyzes were performed using StatView-5 software (SAS Institute, Cary, NC, USA). Statistical values indicated for *p* values < 0.05.

## 5. Patents

EP19218191.5—Detergent-free decellularized extracellular matrix preparation method and bioinks for 3D printing—European Patent Application;

PCT/IB2020/056856—Detergent-free decellularized extracellular matrix preparation method and bioinks for 3D printing—International Patent Application.

## Figures and Tables

**Table 1:** *p*-values obtained for the results presented in Figure 3.

Variants	*p*-Value
CUT/PBS/24 °C, GROUND/PBS/4 °C	<0.0001
CUT/PBS/4 °C, GROUND/PBS/4 °C	<0.0001
CUT/WA/24 °C, GROUND/PBS/4 °C	<0.0001
CUT/WA/4 °C, GROUND/PBS/4 °C	<0.0001
GROUND/PBS/24 °C, GROUND/PBS/4 °C	<0.0001
GROUND/PBS/4 °C, GROUND/NH_3_ · H_2_O/24 °C	<0.0001
GROUND/PBS/4 °C, GROUND/NH_3_ · H_2_O/4 °C	<0.0001

**Table 2:** *p*-values obtained for the results presented in Figure 5.

Variants	*p*-Value
GROUND/PBS/4 °C, GROUND/NH_3_ · H_2_O/4 °C	0.0030
GROUND/PBS/4 °C, GROUND/NH_3_ · H_2_O/24 °C	0.0467
CUT/NH_3_ · H_2_O/4 °C, GROUND/PBS/4 °C	0.0100
CUT/NH_3_ · H_2_O/24 °C, GROUND/PBS/4 °C	<0.0001
CUT/PBS/24 °C, GROUND/PBS/4 °C	0.0019

## Data Availability

Not applicable.
